# Knowledge, Attitude, and Practice of Over-the-Counter Drug Use Among Medical Students in Fakeeh College: A Cross-Sectional Study

**DOI:** 10.7759/cureus.83878

**Published:** 2025-05-11

**Authors:** Ahmed M Faheem, Maha S Bamatraf, Shorouq M Mohammed, Nourah A Al Ghamdi, Reem N Rayani, Maniah M Alharbi

**Affiliations:** 1 Medical Biochemistry, Fakeeh College for Medical Sciences, Jeddah, SAU; 2 Medicine, Fakeeh College for Medical Sciences, Jeddah, SAU

**Keywords:** fakeeh college, jeddah, kap, medical students, otc drugs

## Abstract

Background

Over-the-counter (OTC) drugs are medicines sold without a medical prescription to treat common and temperate medical conditions. Unfortunately, the misconception that OTC drugs are devoid of any harm to users has become established as a commonly held belief. While it is true that most of them are relatively safe, if administered in moderation, misuse is usually associated with the intake of excessive amounts and is burdened with life-threatening consequences. This study aimed to assess the knowledge, attitude, and practice (KAP) of OTC medication use and related factors among medical students at Fakeeh College for Medical Sciences in Jeddah, Saudi Arabia.

Subject and methods

This cross-sectional study was conducted among medical students at Fakeeh University in Jeddah, Saudi Arabia. A self-administered questionnaire was distributed among medical students using an online platform. The questionnaire includes socio-demographic characteristics and a questionnaire to assess OTC drugs' knowledge, attitude, and practice.

Results

Of the 349 medical students, 86.5% were female students, and 52.4% were aged between 21 and 23 years old. The rate of OTC drugs used among medical students was 75.9%. Students who believed that OTC drugs are approved for self-care and those who experienced side effects due to OTC drugs were the significant users of OTC drugs. Approximately 25.5% had positive attitudes toward OTC drugs, 61.6% were neutral, and only 12.9% had negative attitudes. Senior medical students who enrolled in medicine demonstrated better attitudes toward OTC drugs than the rest of the students.

Conclusion

This study supports the literature that there is a growing number of medical students who are using OTC drugs. Regardless of their safety and efficacy, students demonstrated an optimistic attitude toward them. More efforts are needed to increase the knowledge of students about the harmful effects of the excessive use of OTC medication.

## Introduction

Over-the-counter (OTC) medicines are medicines that may be sold directly to a consumer without a prescription from a physician, as compared to prescription drugs, which are dispensed only to consumers possessing a valid prescription [1]. OTC drugs are medicines sold without a medical prescription to treat common and temperate medical conditions [2]. OTC drugs fall into the following ten groups, according to the WHO Anatomical Therapeutic Chemical (ATC) classification: dermatologicals, cough-and-cold remedies, antacids, analgesics, laxatives, antithrombotic agents, antihistamines, throat preparations, nose preparations, and antidiarrheals.

Globally, many studies have reported the prevalence of self-medication among healthcare professionals in both developing and developed countries [3,4].

A cross-sectional study conducted at Aga Khan University in Karachi, Pakistan showed that the prevalence of self-medication was 76% [5]. The study also revealed that the most commonly used medicines were analgesics (88.3%), antipyretics (65.1%), and antibiotics (35.2%). Similar findings were also reported by studies conducted in Serbia and India in which the total prevalence of self-medication was 79.9% and 78.6%, respectively [6,7]. A more profound outcome was reported by a study conducted in Kuwait, in which the overall prevalence of self-medication was 97.8% [8].

A variety of reasons were given as the common motives to practice self-medication, the most common being prior experience and using it in treatment of mild symptoms. In many of the studies, OTC drugs were commonly used, though some of the studies have also reported the use of prescription-only drugs which are dangerous without professional counseling [9].

Self-medication among healthcare professional students, especially medical students, is increasingly worrying because of factors such as academic stress, convenience, and lack of time. Research indicates that there is a considerable variation in students' understanding, attitudes, and behaviors concerning self-medication and the adverse drug reactions that can result from it [10-12].

Few studies have been conducted regarding self-medication. One systematic review revealed that the prevalence of self-medication practice in Ethiopia was between 12.8% and 77.1%, with an average of 36.8% [13].

Other studies conducted in Addis Ababa and Mekelle showed that the prevalence of self-medication was 62.7% and 43.24%, respectively. It was also reported that nearly half of the respondents (47.3%) did not know the medication classification of OTC and prescription-only drugs [14]. However, various studies have been conducted in different countries but never localized at Fakeeh College for Medical Sciences in Jeddah, Saudi Arabia. The aim of this study was to assess the knowledge, attitude, and practice (KAP) of OTC medication use and related factors among medical students at Fakeeh College for Medical Sciences in Jeddah, Saudi Arabia.

## Materials and methods

Setting

This is an epidemiological, cross-sectional study, executed from September 2023 till February 2024 at Fakeeh College for Medical Sciences (FCMS) among medical students in Jeddah, Saudi Arabia (KSA).

Design

The sample size was 347 medical students from the FCMS enrolled in this study. The sample size was measured using the Epi-Info CDC software calculator (Centers for Disease Control and Prevention, Atlanta, Georgia) [15]. The data was collected with a pre-tested validated questionnaire, and it was distributed online [16]. The study population consisted of all healthcare students studying at Fakeeh College of Medical Sciences. Participants were randomly chosen from all years of the healthcare field. All healthcare students from different fields, genders, and nationalities studying at Fakeeh College were included, and students who refused to participate in the research were excluded.

Data questionnaire

The questionnaire is a multidisciplinary piece composed of four different parts (see Appendices). The first part included participants' sociodemographic characteristics, whereas the second part included knowledge of the study participants towards OTC Medication. The third part included the attitude of the respondents towards OTC medication use, and the fourth part consisted of the OTC drug practice-related characteristics of the study participants. Data was entered through a Google Form.

The attitude toward OTC drugs has been assessed using an eight-item questionnaire, with 5-point Likert Scale categories ranging from "strongly disagree" coded with 1 to "strongly agree" coded 5 as the answer option. A negative question has been re-coded inversely to avoid bias in the score. The total attitude score has been calculated by adding all eight items. Scores ranging from 8 to 40 points have been achieved. The higher the score, the higher the attitude toward OTC drugs. By using (50%) and (75%) as cutoff points to determine the level of attitude, medical students were considered as having a negative attitude if the score was below 50%, 50% to 75% were considered as a neutral attitude, and above 75% were considered as having positive attitude levels.

Data analysis

Descriptive statistics were calculated to present numbers and percentages (%) for categorical variables, while means and standard deviations were used to summarize all continuous variables. The attitude score was compared to the socio-demographic characteristics, knowledge, and practice towards OTC using the Mann-Whitney Z-test and the Kruskal-Wallis H-test. The normality test was performed using the Shapiro-Wilk test and Kolmogorov-Smirnov test. Based on the results, the attitude followed the abnormal distribution; therefore, the non-parametric tests were applied. Also, the relationship between the use of OTC drugs according to the socio-demographic characteristics and the knowledge of OTC drugs has been examined using the chi-square test. A P-value of 0.05 was considered statistically significant. The data were analyzed using IBM SPSS Statistics for Windows, Version 26 (Released 2019; IBM Corp., Armonk, New York, United States).

## Results

This study enrolled 349 medical students. As described in Table 1, 183 (52.4%) were aged between 21 and 23 years old, with the majority being female, 302 (86.5%). Nearly all were single, 327 (93.7%). The most commonly chosen field of study was medicine 225 (64.5%). Additionally, 95 (27.2%) were at second-year levels.

**Table 1:** Socio-demographic characteristics of the medical students (n=349) N: Number of individuals; %: percentage

Study variables	N (%)
Age group	
18 – 20 years	97 (27.8%)
21 – 23 years	183 (52.4%)
24 – 26 years	48 (13.8%)
>26 years	21 (06.0%)
Gender	
Male	47 (13.5%)
Female	302 (86.5%)
Marital status	
Single	327 (93.7%)
Married	20 (05.7%)
Divorced	02 (0.60%)
Academic field of study	
Medicines	225 (64.5%)
Nursing	79 (22.6%)
Laboratory science	20 (05.7%)
Pharmacy	25 (07.2%)
Year of Study	
First Year	27 (07.7%)
Second Year	95 (27.2%)
Third Year	60 (17.2%)
Fourth Year	60 (17.2%)
Fifth Year	42 (12.0%)
Sixth Year	65 (18.6%)

Regarding students' knowledge about OTC medications (Table 2), 186 (53.3%) of the students believed that medicine always needed a prescription from the doctor. Students who thought that OTC drugs were effective and safe constituted 129 (37%). According to students' knowledge, OTC drugs were for the treatment of minor illnesses and injuries, 233 (66.8%). Students who knew that OTC drugs are approved for self-care were 208 (59.6%). Only 70 (20.1%) would use OTC drugs after the expiry date. Most of the students believed that OTC drugs can sometimes cause side effects (246; 70.5%). The most common population group that should be cautioned about taking OTC medication was pregnant women, 259 (74.2%). If one experienced side effects, the most common action to be taken was to cease taking the medication immediately, 250 (71.6%). In addition, 141 (40.4%) of the students believed that all OTC drugs can be taken together with prescribed drugs.

**Table 2:** Assessment of knowledge toward OTC medications (n=349) N: Number of individuals; %: percentage; OTC: over-the-counter

Statement	N (%)
Medicines are always used on the prescription of a doctor	
Yes	186 (53.3%)
No	153 (43.8%)
I don't know	10 (02.9%)
All OTC drugs are safe and effective	
Yes	129 (37.0%)
No	180 (51.6%)
I don't know	40 (11.5%)
OTC drugs are usually used for treating diseases like:	
Chronic Illnesses	70 (20.1%)
Minor Illnesses and Injuries	233 (66.8%)
I don't know	46 (13.2%)
OTC drugs are approved for self-care	
Yes	208 (59.6%)
No	75 (21.5%)
I don't know	66 (18.9%)
OTC drugs could be used after their expiry date	
Yes	70 (20.1%)
No	241 (69.1%)
I don't know	38 (10.9%)
OTC drugs can cause side effects	
Sometimes cause side-effects	246 (70.5%)
Mostly cause side-effects	69 (19.8%)
Never cause side-effects	18 (05.2%)
I don't know	16 (04.6%)
While using OTC drugs, caution should be taken mostly during ^†^	
Pregnancy	259 (74.2%)
Lactation	200 (57.3%)
Elderly	166 (47.6%)
Children	220 (63.0%)
Adolescent/Middle Adults	84 (24.1%)
If suspected side-effect(s) are seen, then one should ^†^	
Immediately stop using the drug	250 (71.6%)
Take a low dose until side effects subside	76 (21.8%)
Continue taking the drug regardless of the side effects	38 (10.9%)
Report to a Doctor or Pharmacist	209 (59.9%)
Others	03 (0.90%)
All OTC drugs, when taken along with the prescribed drug, are safe	
Yes	141 (40.4%)
No	129 (37.0%)
I don't know	79 (22.6%)

When examining the attitude toward OTC medications (Table 3), it can be observed that the top three statements with the highest ratings were "OTC drugs are not affected by storage conditions like temperature, moisture, and direct sunlight" (mean score: 3.72), "It is appropriate to seek a pharmacist's advice when someone has OTC medicines that he/she has never used before" (mean score: 3.60) and "It is appropriate to treat minor-ailments like a common cold with OTC medications" (mean score: 3.42). Accordingly, based on eight attitude items, the total mean attitude score was 26.5 (SD 5.76), with negative, neutral, and positive attitudes constituting 12.9%, 61.6%, and 25.5%, respectively.

**Table 3:** Assessment of attitude toward OTC medications (n=349) Response has a range from "strongly disagree" coded with 1 to "strongly agree" coded with 5. * Reverse-coded statement. OTC: Over-the-counter

Statement	Mean ± SD
OTC drugs are not affected by storage conditions like temperature, moisture, and direct sunlight *	3.72 ± 1.22
It is appropriate to seek a pharmacist's advice when someone has OTC medicines that he/she has never used before	3.60 ± 1.34
It is appropriate to treat minor-ailments like a common cold with OTC medications	3.42 ± 1.19
Is there a need to consult a pharmacist for dispensing and using OTC medications?	3.37 ± 1.14
OTC drugs can modify or alter the action of another drug	3.34 ± 1.15
When someone goes to a pharmacy for OTC medication, he/she should bring all medications he/she is currently taking	3.20 ± 1.23
OTC drugs are cheaper and more convenient	3.05 ± 1.11
It is okay to share OTC medications with others	2.75 ± 1.19
Total attitude score (mean ± SD)	26.5 ± 5.76
Level of attitude	
Negative	45 (12.9%)
Neutral	215 (61.6%)
Positive	89 (25.5%)

**Figure 1:**
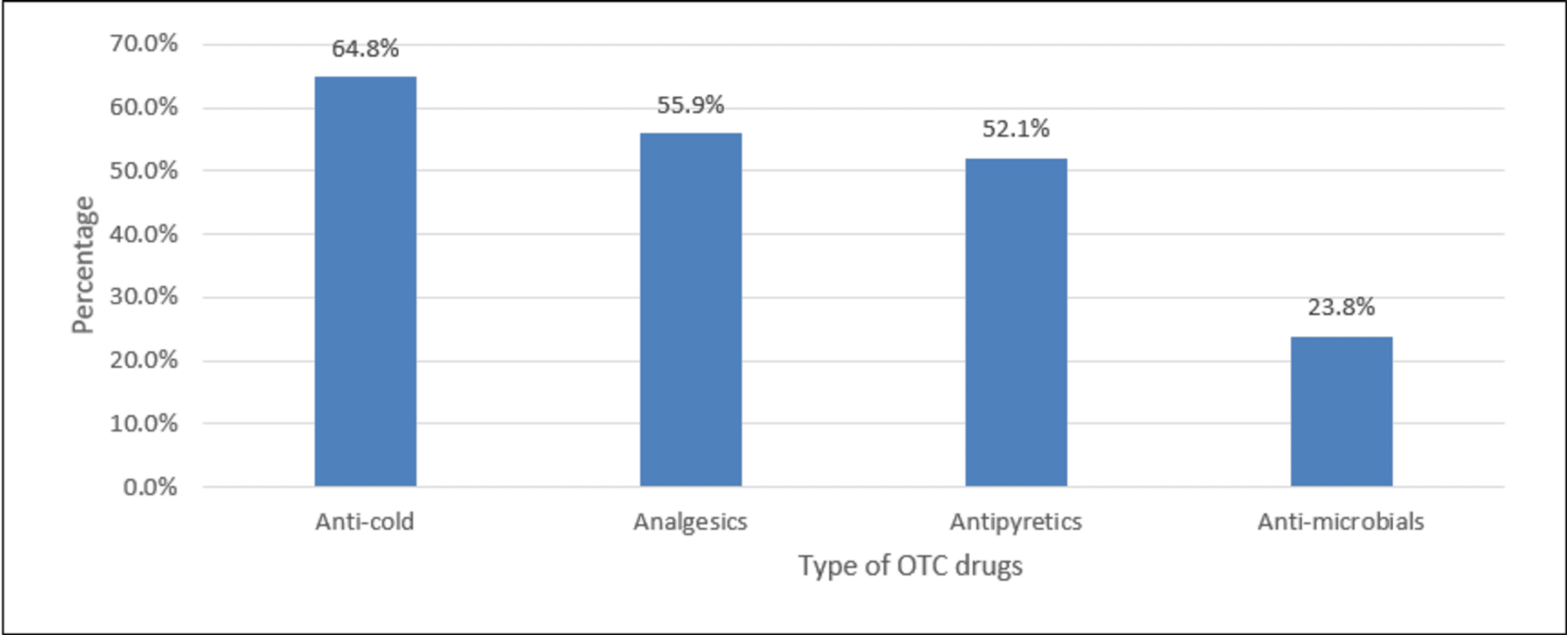
Type of OTC drugs %: percent, polls represent the percentage of responses OTC: Over-the-counter

As illustrated in Figure 1, the most common types of OTC drugs were anti-cold (64.8%) and analgesics (55.9%).

**Figure 2:**
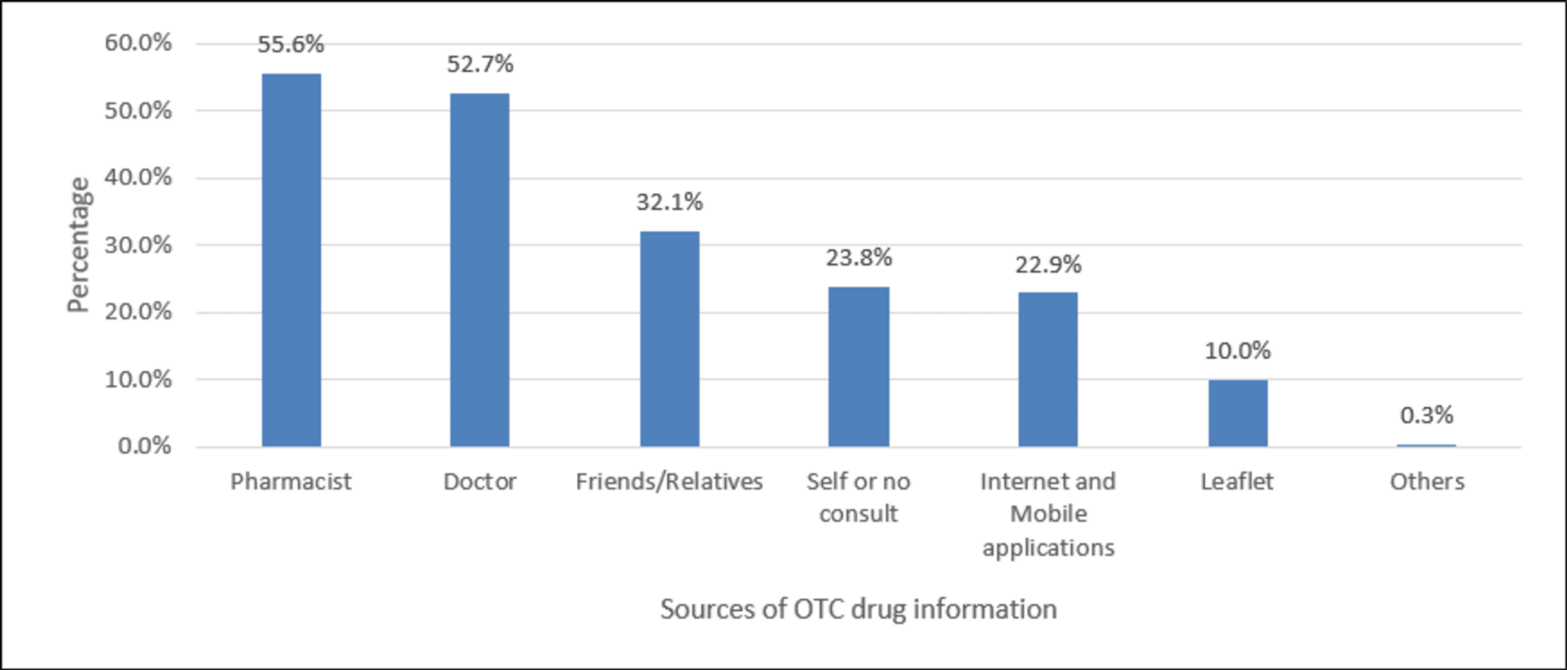
Sources of OTC drug information %: percent, polls represent the percentage of responses OTC: Over-the-counter

Figure 2 showed that the most common source of OTC drug information was a pharmacist (55.6%), followed by a doctor (52.7%) and friends/relatives (32.1%).

**Figure 3:**
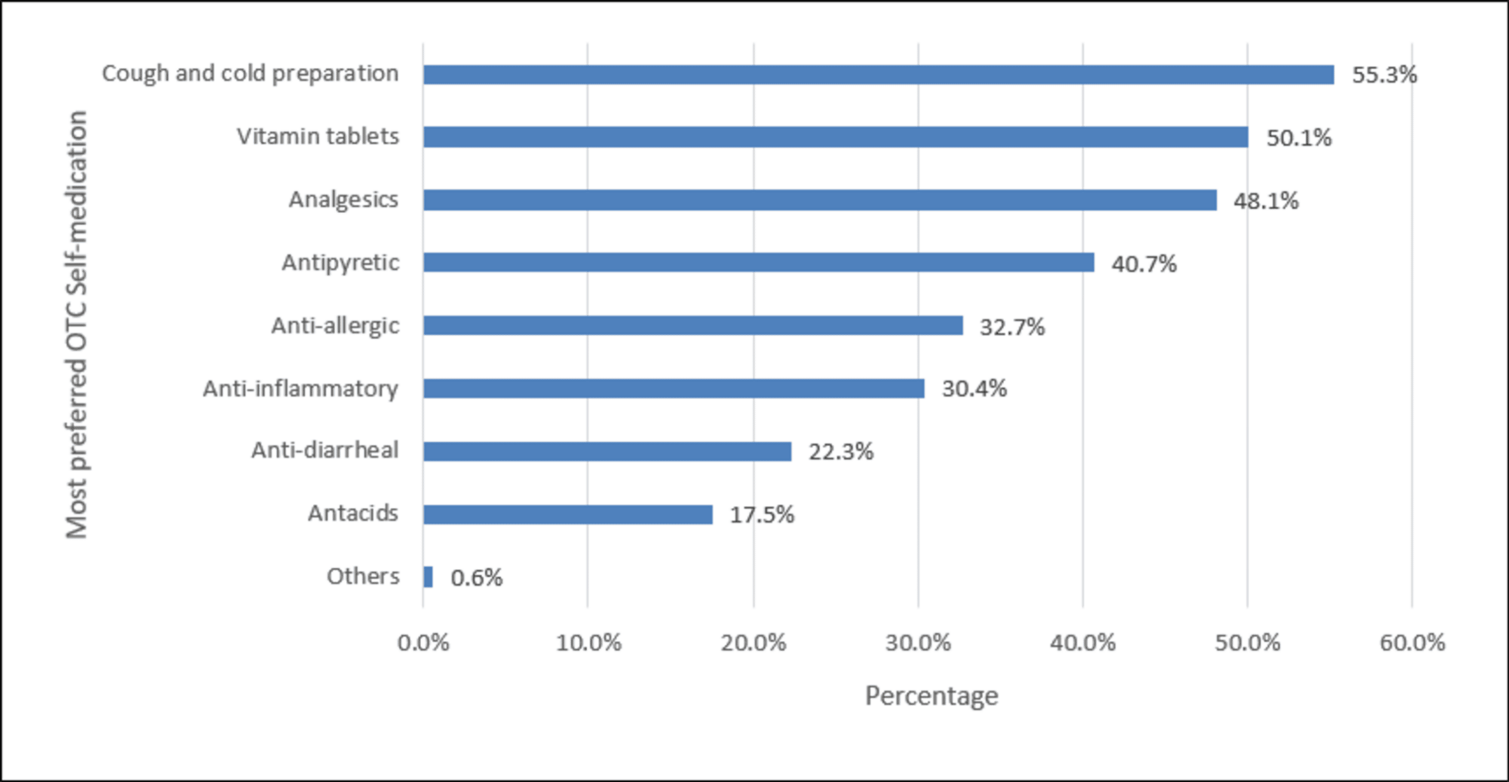
Most preferred OTC self-medication %: percent, polls represent the percentage of responses OTC: Over-the-counter

In Figure 3, the commonly preferred OTC self-medication was cough and cold preparation (55.3%), followed by vitamin tablets (50.1%) and analgesics (48.1%).

Regarding the assessment of practice toward OTC medications (Table 4), it was revealed that 265 (75.9%) were regularly taking OTC drugs. One hundred and ninety-nine (57%) usually take OTC drugs for minor symptoms, while the most common reason for choosing OTC drugs is that they are accessible (204; 58.5%). The proportion of students who experienced side effects after taking OTC drugs was 133 (38.1%). Students who regularly read OTC drug instructions constitute 139 (39.8%), while those who regularly check expiry dates were 190 (54.4%). More than half (188; 53.9%) would go to a healthcare facility if an OTC medication does not work for them. The most common action to be taken if OTC drugs show a change in shape, color, or odor is to discard the drug immediately, 273 (78.2%). In addition, 181 (51.9%) preferred to store OTC drugs in a medicine box.

**Table 4:** Assessment of practice toward OTC medications (n=349) * Variable with multiple response answers N: numbers; %: percentage; OTC: over-the-counter

Statement	N (%)
Have you ever practiced medication use with OTC drugs?	
Yes	265 (75.9%)
No	84 (24.1%)
When did you consume OTC drugs? *	
When symptoms are minor and manageable	199 (57.0%)
Whenever I feel sick	174 (49.9%)
When I can't visit a doctor	139 (39.8%)
Others	03 (0.90%)
Common reason(s) for using OTC drugs is *	
Time-saving	193 (55.3%)
Low cost	167 (47.9%)
Safe and well tolerable	114 (32.7%)
Easy accessibility	204 (58.5%)
Others	05 (01.4%)
Have you ever experienced adverse effects from OTC drugs?	
Yes	133 (38.1%)
No	216 (61.9%)
How often do you read the instructions on the drug's label before use?	
Always	139 (39.8%)
Occasionally	100 (28.7%)
Rarely	85 (24.4%)
Never	25 (07.2%)
How often do you check the expiry date?	
Always	190 (54.4%)
Occasionally	83 (23.8%)
Rarely	50 (14.3%)
Never	26 (07.4%)
What do you do in case OTC drugs do not work well (not cure)? *	
I double the dose	110 (31.5%)
I change to another powerful over-the-counter drug	121 (34.7%)
I go to a health facility	188 (53.9%)
Others	03 (0.90%)
What do you do if OTC drugs show a change in shape, color, and or odor? *	
Immediately discard the drug	273 (78.2%)
Continue using till it expires	68 (19.5%)
Continue using even if after it expires	35 (10.0%)
Others	01 (0.30%)
Where do you usually store OTC drugs? *	
Medicine box	181 (51.9%)
Bedroom/open in the table	134 (38.4%)
Refrigerator	151 (43.3%)
Kitchen	54 (15.5%)
Bathroom	27 (07.7%)

When measuring the association between the attitude score in relation to the socio-demographic characteristics, the knowledge and practice of OTC drugs (Table 5), it was found that a higher attitude score was more associated with being enrolled in medicines (Z=2.570; p=0.010), being senior students (Z=4.437; p<0.001), those who do not believe that medicines always need doctor's prescription (Z=3.925; p<0.001), those who do not believe that all OTC drugs are safe and effective (Z=3.156; p=0.002), those who were not aware that OTC drugs could be used after its expiry date (Z=5.147; p<0.001), those who were against OTC drugs can be taken along with prescribed drug (Z=5.343; p<0.001), those who were taking OTC drugs (Z=2.563; p=0.010) and those who did not experience side effects from OTC drugs (Z=5.715; p<0.001).

**Table 5:** Association between attitude among the socio-demographic characteristics, the knowledge, and practice of OTC drugs (n=349) § The p-value has been calculated using the Mann-Whitney Z-test. ‡ The p-value has been calculated using the Kruskal-Wallis H-test. ** Significant at p<0.05 level.

Factor	Attitude Score (40) Mean ± SD	Z/H-test	p-value ^§^
Age group			
18 – 20 years	25.5 ± 5.71	4.405	0.111 ^‡^
21 – 23 years	26.7 ± 5.88		
>23 years	27.2 ± 5.39		
Gender			
Male	27.3 ± 6.58	1.522	0.128
Female	26.3 ± 5.62		
Academic field of study			
Medicines	27.1 ± 5.47	2.570	0.010 **
Non-medicine	25.4 ± 6.13		
Academic year level			
Junior students (1^st^ – 3^rd^ year)	25.2 ± 5.94	4.437	<0.001 **
Senior students (4^th^ – 6^th^ year	27.8 ± 5.24		
Medicines are always used on the prescription of a doctor			
Yes	25.4 ± 5.79	3.925	<0.001 **
No	27.7 ± 5.49		
All OTC drugs are safe and effective			
Yes	25.0 ± 6.93	3.156	0.002 **
No	27.6 ± 4.86		
OTC drugs are approved for self-care			
Yes	26.4 ± 6.40	1.411	0.158
No	25.7 ± 5.23		
OTC drugs could be used after their expiry date			
Yes	22.4 ± 7.39	5.147	<0.001 **
No	27.5 ± 4.68		
All OTC drugs, when taken along with the prescribed drug, are safe			
Yes	24.1 ± 6.38	5.343	<0.001 **
No	28.1 ± 4.79		
Have you ever practiced medication use with OTC drugs?			
Yes	26.7 ± 5.93	2.563	0.010 **
No	25.7 ± 5.13		
Have you ever experienced adverse effects from OTC drugs?			
Yes	23.9 ± 6.54	5.715	<0.001 **
No	28.0 ± 4.60		

When measuring the relationship between the use of OTC drugs with regard to the socio-demographic characteristics, the knowledge, and other related practices of OTC drugs (Table 6), it was observed that students who knew that OTC drugs are approved for self-care (p=0.041) and those who experienced adverse effects from OTC drugs (p=0.005) were more likely to use OTC drugs.

**Table 6:** Relationship between the use of OTC drugs among the socio-demographic characteristics, the knowledge, and practice of OTC drugs (n=349) § The P-value has been calculated using the chi-square test. ** Significant at p<0.05 level. OTC: Over-the-counter

Factor	Use of OTC drugs		P-value ^§^
	Yes N (%) ^(n=265)^	No N (%) ^(n=84)^	
Age group			
18 – 20 years	76 (28.7%)	21 (25.0%)	0.534
21 – 23 years	140 (52.8%)	43 (51.2%)	
>23 years	49 (18.5%)	20 (23.8%)	
Gender			
Male	37 (14.0%)	10 (11.9%)	0.630
Female	228 (86.0%)	74 (88.1%)	
Academic field of study			
Medicines	175 (66.0%)	50 (59.5%)	0.277
Non-medicine	90 (34.0%)	34 (40.5%)	
Academic year level			
Junior students (1^st^ – 3^rd^ year)	138 (52.1%)	44 (52.4%)	0.961
Senior students (4^th^ – 6^th^ year	127 (47.9%)	40 (47.6%)	
Medicines are always used on the prescription of a doctor			
Yes	136 (52.5%)	50 (62.5%)	0.117
No	123 (47.5%)	30 (37.5%)	
All OTC drugs are safe and effective			
Yes	105 (43.8%)	24 (34.8%)	0.183
No	135 (56.3%)	45 (65.2%)	
OTC drugs are approved for self-care			
Yes	168 (76.4%)	40 (63.5%)	0.041 **
No	52 (23.6%)	23 (36.5%)	
OTC drugs could be used after their expiry date			
Yes	56 (23.4%)	14 (19.4%)	0.478
No	183 (76.6%)	58 (80.6%)	
All OTC drugs, when taken along with the prescribed drug, are safe			
Yes	108 (52.2%)	33 (52.4%)	0.977
No	99 (47.8%)	30 (47.6%)	
Have you ever experienced adverse effects from OTC drugs?			
Yes	112 (42.3%)	21 (25.0%)	0.005 **
No	153 (57.7%)	63 (75.0%)	

## Discussion

This study investigated medical students' KAP about OTC drugs and determined the factors that influence them the most. The results of this study would be an important addition to the literature, given that self-medication is widely practiced throughout the globe. Hence, increased awareness of OTC misuse is crucial among users.

Knowledge about OTC drugs

Consulting the knowledge of medical students about OTC drugs was inadequate. Only 129 (37%) were confident that OTC drugs are safe and effective. Also, we noted that even though the majority were aware of OTC drugs' adverse effects, some students (70; 20.1%) indicated that they could be consumed even after the expiry date, while only 141 (40.4%) believed that OTC medications are safe even when consumed along with the prescribed drug. In India [16], pharmacy students' knowledge regarding the efficacy of OTC drugs was higher. Approximately 87.1% believed that OTC was safe and that the use of OTC drugs was mainly due to economic problems and lack of time to visit physicians. In our study, being accessible and time-saving were the main reasons for choosing OTC drugs. On the other hand, in Pakistan [5], most university students believed that self-medication could be detrimental to health and practice.

Almost 83% thought about consulting a doctor before taking new medicine, which was supported by the study done in Malaysia [10]. However, in a study done by Thadani et al. [15], the lack of understanding of drugs and their side effects was identified as the main reason for not practicing self-medication. In contrast, studies done by Sharma et al. [17] as well as Elbur et al. [11] showed satisfactory knowledge about self-medication among the public.

Sources of OTC drug information

The sources of information are one of the cores of knowledge. In this study, the sources of OTC drug information were mainly from the pharmacist (55.6%) and the doctor (52.7%). Other sources, such as friends and relatives, self-experience, internet and mobile applications, and leaflets, were rated less. There are conflicting reports regarding the sources of information related to OTC medications. For instance, Bollu et al. [16] reported that pharmacy students' most common source of information was family and friends (48%), followed by the pharmacist (31.7%), while Beyene et al. [18] cited reading material (56.3%) and pharmacist (43.8%), which was in accordance with the report of Abay and Amelo [3]. Current research suggests that the use of over-the-counter drugs is prevalent, underscoring the need to raise awareness and educate students about the benefits and risks associated with self-medication [19].

Attitude toward OTC drugs

Medical students seem to have optimistic attitudes toward OTC drugs. Results from our study revealed that the majority (61.6%) were deemed to have a neutral attitude, 25.5% were positive, and only 12.9% were negative (mean score: 26.5 out of 40 points). This almost mirrored the report of Bekele et al. (2020) [14]. The mean attitude toward OTC drugs among students was 26.6 out of 40 points, which is likely to be in the neutral category.

Significant factors of attitude

In this study, we identified several factors that could likely influence the attitude toward OTC drugs, including being enrolled in a medicine specialty, being a senior student, believing that medicines are not always based on a doctor's prescription, all OTC drugs are not safe and effective, OTC drugs cannot be used after expiry date, OTC drugs are not safe when taken along with prescribed drug, regular use of OTC medications and did not experience adverse effect from taking OTC drugs. Contradicting these reports, in Brunei [20], a study found no significant relationship between the attitude and the socio-demographic characteristics of the students.

Specific details of attitude

When examining attitude details, we noticed that the ratings were higher in 7 out of 8 items, with mean scores ranging from 3.05 to 3.72 points (out of 5 points). Only the statement about "sharing OTC medications with others" did not reach 3 points (mean score: 2.75). This low rating is understandable, students may be hesitant to share non-prescribed drugs with someone due to varying reasons that may be beyond the scope of this study. The healthcare students in Riyadh [1] demonstrated conflicting attitudes toward OTC drugs. Generally, students were seen to have a positive attitude towards pharmacists when providing consultations; however, most of them showed a negative attitude about the pharmacist's advice on nutritional supplements. In contrast, among healthcare and pharmacy students in India [19], a deficient attitude toward OTC drugs was seen in most students. For instance, a vast majority of the students were not reading the label content of the medicine (75%), resulting in a lack of knowledge of the common side effects and contraindications of the drugs, with only 22% aware of the drugs' side effects. Our respondents also exhibited these attitudes, as only 39.8% regularly read the drug's label before use.

Practice toward OTC drugs

There was a predominant use of OTC drugs among medical students. More than three-quarters were regularly taking OTC drugs. Multiple studies have documented high consumption rates of OTC medications among students, ranging from 38% to 98% [8,16,17,19,21]. However, a study done by Thadani et al. [15] reported that most students were not frequently doing self-medication (79.4%), with only 11.3% usually practicing it, but 9.3% were not practicing self-medication at all. A study conducted by Al-Hussaini et al. [8] reported the highest prevalence of self-medication at 97.8%.

Significant factor of practice

This study found no significant relationship between the use of OTC drugs and the demographic variables of the medical students (p>0.05). However, we noted that the use of OTC drugs was more prevalent among those who knew that OTC drugs are approved for self-care (p=0.041) and those with previous experience of OTC drug side effects (p=0.005). This is comparable to the previous study by Abay and Amelo [3]. Even though they found a significant association between self-medication and the year of study, a lack of evidence was seen for the association between self-medication in terms of medical versus nonmedical, males versus females, and the type of school. Opposing these results, Lukovic et al. [6] detected an association between self-medication and home pharmacies, lower level of father education, female gender, older age, and physical activities.

Specific details of practice

Most of our respondents were using OTC medication because of minor symptoms or whenever they felt ill. Being accessible and time-saving were identified as the most frequent reasons for choosing OTC drugs. We also observed that even though their practice for adverse effects was seen to be good, their action when OTC drugs do not work well seems conflicting. Only 53.9% had the correct practice to consult a doctor when OTC failed to improve their condition. In Egypt [4], nearly two-thirds discontinued the medication if they felt it was not improving, and 60% chose to increase the dose without medical advice. Only 14.4% followed what was written in the prescription. This has been supported by the study in Pakistan [9], wherein 43% of the respondents said they modified the prescribed medicine regimen, and 61.9% ceased taking medications without visiting a doctor.

Future prospects and limitations 

Self-medication among healthcare professional students, especially medical students, is increasingly worrying because of factors such as academic stress, convenience, and lack of time. Research indicates that there is a considerable variation in students' understanding, attitudes, and behaviors concerning self-medication and the adverse drug reactions that can result from it.

While this study offers insightful information, there are some important limitations to be aware of. There are limitations in establishing causality or tracking changes over time with the cross-sectional approach. In addition, response bias could be introduced by the study's dependence on self-reported data.

## Conclusions

Despite gaps in knowledge and attitude, non-prescribed medication practices were common among medical students enrolled at Fakeeh University. However, a better attitude toward OTC drugs was seen more frequently among senior medical students who had a better understanding and practice of the use of OTC drugs. Consistent with the literature, this study supports the fact that the use of OTC drugs was prevalent even among medical students. Hence, continuous awareness is crucial to educate students about the pros and cons of OTC medications. Pharmacists and other healthcare professionals may have vital roles in delivering the appropriate information about the advantages and disadvantages of self-medication.

## Appendices

Survey questionnaire

This questionnaire is prepared to assess knowledge, practice, and attitude toward medication use and related adverse effects among medical students at Fakeeh University. The response that you willingly give will facilitate the completion of the study. The research is purely for academic purposes. Your genuine response to these questions will help in the correct finding of the study. 

Are you willing to participate?      A.  Yes                        B.  No

Section A: Sociodemography

Instruction: Circle, put a tick/cross, or write the answer that corresponds with your best answer.

**Table 7:** Sociodemographic questions

No	Socio-demographic questions					Answer
1.	Sex			1. Male 2. Female		
2.	Age in years			-------------- in years		
3.	What is your marital status?		1. Single (never married) 2. Married 3. Divorced 4. Widowed			
4.	What is your academic field of study?				1. Medicine 2. Pharmacy	
5.	What is your year of study?				3^rd^ year 4^th^ year 5^th^ year Intern Doctor	
6.	What is your religion?	1. Orthodox 2. Muslim 3. Protestant 4. Other specify................				
7.	What is the average family income per month? ──── Birr (on average # per month)					
8.	What is your average money monthly revived from family others? ________ Birr (on average # per month)					

Section B: Knowledge

Instruction: Circle your answers on the choices provided at the right side

**Table 8:** Questions about knowledge OTC: Over-the-counter

No	Questions about knowledge	Answer		
1.	Medicines are always used on the prescription of a doctor. 1. Yes 2. No 3. Don’t know			
2.	All over-the-counter drugs are safe and effective. 1. Yes 2. No 3. Don’t know			
3.	Over-the-counter drugs are usually used for treating diseases like:			
	1. Chronic illnesses 2. Minor illnesses and injuries 3. Don’t know			
4.	Over-the-counter drugs are approved for self-care.			
	1. Yes 2. No 3. Don’t know			
5.	Which of the following drugs fall under OTC drugs? (multiple answers)			
	a. Antipyretics b. Anti-cold c. Analgesics d. Anti-microbials			
6.	Over-the-counter drugs could be used after their expiry date.			
	1. Yes 2. No 3. Don’t know			
7.	Over-the-counter drugs can: 1. Sometimes cause side-effect(s) 3. Never cause side-effect(s) 2. Mostly cause side-effect(s) 4. Don’t know			
8.	While using over-the-counter drugs, caution should be taken mostly in: (multiple answers)			
	a. Pregnancy b. Lactation	c. Elderly d. Children		e. Adolescent/middle adults
9.	If suspected side-effect(s) are seen, then one should: (multiple answers)			
	a. Immediately stop using the drug b. Take low dose until side effect(s) subside c. Continue taking the drug regardless the side effect(s)		d. Report to a Doctor or Pharmacist e. Other, please specify ---------------------------	
10.	All over-the-counter drugs when taken along with prescribed drug are safe.			
	1. Yes 2. No 3. Don’t know			

Section C: Attitude

Instruction: For the following section, the choices are represented by numbers in which 1=Strongly disagree, 2=Disagree, 3=Neither agree or disagree, 4=Agree, and 5=Strongly agree. Circle or tick √ ONE answer that best matches your general opinion.

**Table 9:** Statements about attitude OTC: Over-the-counter

No	Statements			Strongly Disagree	Disagree	Neither Agree or Disagree	Agree	Strongly Agree
1.	Over the Counter drugs are cheaper and convenient.			1	2	3	4	5
2.	Have you ever given your OTC medication to others?			1	2	3	4	5
3.	Pain killers when taken on an empty stomach does not cause gastritis			1	2	3	4	5
4.	Over the Counter drugs can modify or alter the action of another drug.			1	2	3	4	5
5.	You would seek a pharmacist advice when you have OTC medicines that you were never used before.			1	2	3	4	5
6.	Pain killers when taken on an empty stomach does not cause gastritis.			1	2	3	4	5
7.	Over the Counter drugs are not affected by storage conditions, like temperature, moisture and direct sunlight.			1	2	3	4	5
8.	When you visit your pharmacist, will you bring all medications you are currently taking?			1	2	3	4	5
9.	Is there a need to consult a pharmacist for dispensing and using OTC medications?			1	2	3	4	5
10.	When you have a cold, will you seek OTC medications in the community pharmacy?			1	2	3	4	5
12.	Do you favor of OTC self-medication use? 1. Yes (skip to12.1) 2. No (skip to12.2)							
	12.1. What are your reasons in favor of OTC self-medication? Multiple answers							
	1. Time-saving 2. No need to visit doctor for minor illness 3. Economical		4. Quick relief 5. Learning opportunity 6. Ease and convenience		7. Crowd avoidance 8. Other, please specify -----------------			
	12.2. What are your reasons against self-medication? Multiple answers							
	1. Risk of adverse drug reactions 2. Risk of using wrong drugs	3. Risk of wrong use of drugs 4. Risk of missing the diagnosis		5. Risk of drug dependence 6. Other, please specify ----------------				

Section D: Practice

Instruction: Circle your answers on the choices provided at the right side.

**Table 10:** Questions regarding practice

No	Questions regarding Practice												Answer		
1.	Have you ever practiced medication use with over-the-counter drugs?												1. Yes (Skip to Q. 2) 2. No (question ends)		
2.	With whom did you consult before using over-the-counter drugs? (multiple answers)														
	1. Pharmacist 2. Doctor	3. Friends/relatives 4. Leaflet					5. Internet and mobile applications 6. Self or No consult 7. Other, please specify-----------								
3.	When did you consume over-the-counter drugs? (multiple answers)														
	1. When symptoms are minor/manageable 2. Whenever I feel sick								3. When I can't visit doctor 4. Other, please specify--------------------						
4.	Common reason(s) for using over-the-counter drugs is: (multiple answers)														
	1. Time saving 2. Low cost		3. Safe and well tolerable 4. Easy accessibility									5. Other, please specify --------------------------------------------------			
5.	Which categories of medications are mostly preferred by you for self- medication? (multiple answers)														
	1. Antipyretic 2. Cough and cold preparation 3. Analgesics			4. Anti-inflammatory 5. Anti-diarrheal 6. Antacids							7. Vitamin tablets 8. Anti-allergic 9. Other, please specify -------------------------				
6.	Have you ever taken Over the Counter drug(s) more than the recommended dose?													1. Yes (skip to Q. 7) 2. No (skip to Q. 8)	
7.	Why did you take more than the recommended dose?									-----------------------------------------------					
8.	Have you ever experienced adverse effect from over-the-counter drugs?														1. Yes (skip to Q. 9) 2. No (skip to Q. 12)
9.	What was the side-effect(s)? ------------------------------------------------------------------------------------------------														
10.	After what medication was the side- effect(s) experienced? ------------------------------------------------------														
11.	What did you do after the side effect(s)? ------------------------------------------------------														
12.	How often do you read the instructions on drug’s label before use?														
	1. Always 2. Occasionally 3. Rarely 4. Never														
13.	How often do you check the expiry date?														
	1. Always 2. Occasionally 3. Rarely 4. Never														
14.	What do you do in case Over the Counter drug do not work well (not cure)? (multiple answers)														
	1. I double the dose 2. I change other powerful Over the Counter drug										3. I go to health facility 4. Other, please specify-------------------------				
15.	What do you do, if Over the Counter drugs showed change in shape, color and or odor? (multiple answers)														
	1. Immediately discard the drugs 2. Continue using till it expires					3. Continue using even if after it expires 4. Other, please specify-------------------------									
16.	Where do you usually store over-the-counter drugs? (multiple answers)														
	1. Medicine box 2. Bed room/open in the table				3. Refrigerator 4. Kitchen							5. Bathroom 6. Other, please specify-------------------------			
17.	What is the average number of over-the-counter drugs that you used per month commonly?										____________ (Average no. OTC per month)				
18.	Do you purchased and or used medications in the last two weeks? 1. Yes (Skip to Q.18.1 ) 2. No														
	18.1. How do you get the medication from pharmacy shop?							1. without prescription paper 2. By using prescription paper							
	18.2. Would you write the name of the purchased medication? _____________________________												--------------------------------------------------		
	18.3. What was the name of the illness of the medication used for?												--------------------------------------------------		

                                             Thank you very much for completing this questionnaire!
